# Thoracic shape changes in newborns due to their position

**DOI:** 10.1038/s41598-021-83869-8

**Published:** 2021-02-24

**Authors:** Serena de Gelidi, Andy Bardill, Nima Seifnaraghi, Yu Wu, Andreas Demosthenous, Marika Rahtu, Merja Kallio, Richard Bayford

**Affiliations:** 1 Faculty of Science & Technology, Middlesex University, London, UK; 2 Department of Electronic and Electrical Engineering, University College London, London, UK; 3 PEDEGO Research Unit, Medical Research Center Oulu, University of Oulu, Oulu, Finland; 4 Department of Children and Adolescents, Oulu University Hospital, Oulu, Finland

**Keywords:** Paediatrics, Biomedical engineering

## Abstract

The highly compliant nature of the neonatal chest wall is known to clinicians. However, its morphological changes have never been characterized and are especially important for a customised monitoring of respiratory diseases. Here, we show that a device applied on newborns can trace their chest boundary without the use of radiation. Such technology, which is easy to sanitise between patients, works like a smart measurement tape drawing also a digital cross section of the chest. We also show that in neonates the supine position generates a significantly different cross section compared to the lateral ones. Lastly, an unprecedented comparison between a premature neonate and a child is reported.

## Introduction

Newborns featuring lung immaturity require continuous monitoring and treatment in Neonatal Intensive Care Units (NICU); an intervention that is critical for the survival of preterm babies. An estimated 15 million premature neonates are born each year^1^. Recently, the CRADL project has introduced the continuous assessment of regional lung function using Electrical Impedance Tomography (EIT) technology as supportive care for the most common causes of paediatric respiratory failure (http://cradlproject.org/). As part of the project, a textile electrode patient interface for the neonatal EIT measurement (SenTec AG, CH) has been developed and tested to validate its clinical performance in a multicentre clinical study^2^. Results showed the absence of any discomfort for the patients whilst providing a low contact impedance, which ensures that good quality measurements can be taken. Hence, such technology gives clinicians a novel insight about the effect of the prescribed therapies on newborns’ respiration^3,4^. However, it has been noticed that in some patients the fitting of the device, designed as a belt for the thorax, was not optimal in all lying positions of the babies. The highly compliant chest wall of neonates increases the tendency to chest wall recession particularly for premature babies^5^. Thus, respiratory diseases leading to poor lung compliance makes neonates prone to respiratory failure and the need for mechanical ventilation. Therefore, an investigation about the geometric change of the chest wall was launched as part of the CRADL project, aiming to improve the performance of the device.

Measurements of the newborn chest are not part of the consolidated clinical routine. At birth, the height, the weight and the head circumference are noted by routine. Therefore, to the best of the authors’ knowledge, no study has collected data about the chest perimeter of newborns and its physiological biomechanical behaviour.

Such information is critically important for predicting the fitting of the neonatal belt in patients of different weight and gestational ages. Almost a century ago, Schultz^6^ pointed out that the literature on human foetal growth was not very extensive. Nowadays, whilst it is well known by clinicians that the morphology of the human chest changes significantly from the neonatal to the adult stages, no extant database quantifies this evolution. In the 80s, a rare chest morphology study compared the influence of age and sex on high altitude populations by carrying out anthropometric measurements at the end of a normal expirations in the age range 5–24 years old^7^. Recently, Chang et al.^8^ have presented a non-invasive method to record the shape of the anterior chest wall to treat infants affected by *pectus excavatum*. Although nowadays chest CT and radiographs provide the required details, children are much more radio-sensitive than adults. Hence, they decided to attach a strip of thermal plastic to obtain a permanent contour of the anterior chest wall^8^. The strip solidified after 10 providing a cross-sectional view of the thorax, which was then scanned for subsequent data analysis.

Therefore, there is a clinical need to acquire, without the use of radiation, the torso shape for treating a variety of respiratory diseases, which are particularly critical for infants. The authors have recently presented a wearable device featuring accelerometers that could successfully carry out the acquisition of different shape boundaries in vitro^9^.

The present study aims at applying the developed technology in vivo for an unprecedented collection of neonatal torso shape related measurements performed in real time by means of an electronic measuring belt. Hence, the goal is to quantify, by means of up-to-date technology, the biomechanical changes of the neonatal chest in different lying positions. As a result, the morphological differences observed in a small population of newborns will be available to neonatologists. Such insight will also give a better understanding about the different configurations that any EIT device needs to adapt to. Therefore, the overall objective is to understand how the mechanical aspect of EIT monitoring could be improved for an increased quality of data supplied to clinicians.

## Results

The novel electronic measuring belt was used on 1 child and 31 newborns, 6 of which were in the NICU. It should be noted that patient no. 20 could not be placed in the prone configuration because of a tracheotomy. Similarly, patient no. 31 could not be measured in the prone position as she was intubated at the time of the study, hence it has not been possible to carry out the complete protocol.

**Figure 1:**
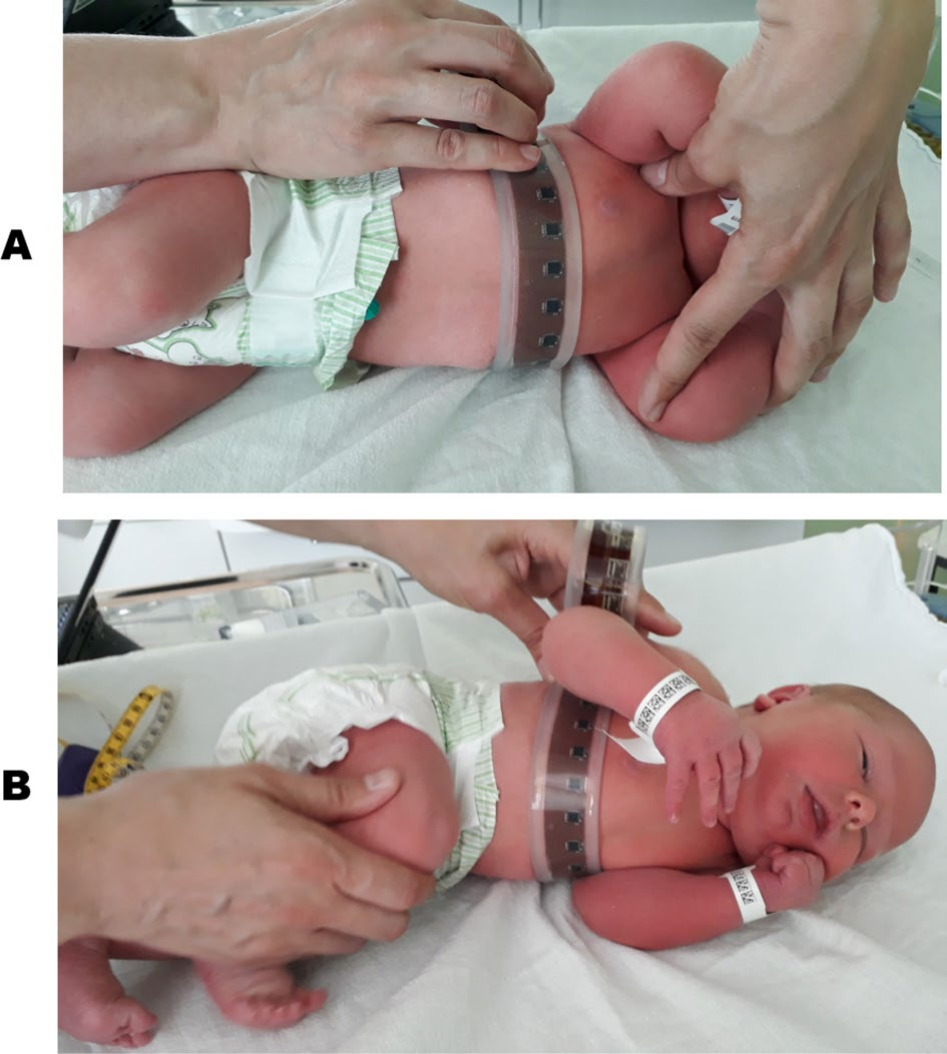
The electronic measuring belt applied on one of the recruited subjects in the maternity ward. According to the protocol, in this shot the clinician was recording the torso shape of the baby lying on his left side with his arms up (**A**) and down (**B**). The photographs are published with permission of both of the baby’s parents.

None of the measurements were interrupted because of the device or because the parents attending the study lacked confidence in the procedure carried out on their babies. No restriction of breathing or skin irritation was observed. The belt was laid on the mattress while each baby was placed on top of it perpendicular to the belt. The belt was wrapped around the chest and held in place by the clinician, as shown in Fig. 1, for less than 5 s whilst the measurement was captured. In order to change the position, the belt was unwrapped, the baby rotated to the next required position and the belt wrapped into position once again for the next measurement to be captured. The study was conducted at a baby-friendly pace, meaning that sleepy participants were faster to move around whilst the more active ones were offered more time to settle in the position. Overall, the study took no more than 5 min to be completed on each subject. The data of the recruited subjects are listed in Table 1. Over 80% of the newborns were born at term, being defined after 37 weeks of Gestational Age. Sixteen of the newborn participants were male and fifteen were female.

**Table 1:** Data of the recruited patients.

Patient	GA	PA	Sex	Ward	Height (cm)	Weight (g)	Head (cm)	Chest (cm)
1	40 0	0 0 17	M	Maternity	50	3600	36	34.5
2	41 6	0 2 9	M	Maternity	50	3720	37	33
3	39 2	0 0 24	F	Maternity	50.5	3650	35	32.5
4	40 3	0 0 19	F	Maternity	50	3592	34.5	33
5	41 1	0 2 17	M	Maternity	50	3872	35.6	34
6	36 4	0 3 4	M	Maternity	50	3292	35	31
7	41 2	0 0 8	F	Maternity	52	3990	33.2	35
8	39 2	0 0 12	M	Maternity	51	3680	36.1	34
9	41 1	0 1 16	M	Maternity	50	3446	35	34.5
10	40 5	0 2 10	M	Maternity	48	3626	36.6	34
11	38 4	0 1 1	M	Maternity	47.5	3060	34	32
12	39 0	0 1 6	M	Maternity	51	3460	35.6	32
13	40 1	0 1 12	M	Maternity	50	3700	35.2	33
14	39 1	0 0 5	F	Maternity	49.5	3160	35	32
15	39 5	0 2 15	F	NICU	52	3905	34.4	35
16	33 6	0 2 13	F	NICU	45	2530	31.9	30
17	33 1	1 0 0	F	NICU	44.5	2380	30	30
18	40 1	1 3 0	F	Maternity	49	4050	37.1	35.5
19	30 1	2 2 0	M	NICU	40	1465	27	24.5
20	38 2	8 3 0	F	NICU	51.8	3796	36.6	33
21	38 4	0 1 15	F	Maternity	49	3477		33.5
22	42 0	2 3 0	M	Maternity	54	4890	37.7	38
23	36 5	0 0 19	F	Maternity	48.5	3200	33.4	32
24	39 4	0 0 20	F	Maternity	50	3380	34.3	33.5
25	38 1	0 1 4	M	Maternity	49.5	3258	33	33
26	39 4	0 1 15	M	Maternity	51	3670	35	35
27	37 6	0 0 16	M	Maternity	48	2795	34.3	30.5
28	39 5	0 0 18	M	Maternity	51	3290	34.8	33
29	37 3	0 0 20	F	Maternity	48	2900	33.9	32
30	41 2	0 0 9	F	Maternity	55	4355	36.7	35.5
31	30 6	4 0 0	F	NICU	41.7	1795	29	27.5

Patient		PA	Sex		Height (cm)	Weight (g)		Chest (cm)
32		6 2	F		130	20000		57

*GA* is the Gestational age (weeks, days), *PA* is the Postnatal age (weeks, days, hours) for the newborns and (years, months) for the child. *Sex* is indicated as *M* for male and *F* for female. *Chest* value reported here was obtained by a standard tape measurement.

The performance of the belt has been analysed by calculating its accuracy and repeatability on the recruited newborns. Since the tape measurement was performed on all subjects in the supine position, this was selected as the gold standard. Hence, the accuracy was calculated as the difference between the mean of the two measurements carried out in supine position and the chest perimeter reported in Table 1. The maximum offset of 1.7 cm was reported for subject number 9, whilst the minimum of 0.5 mm was estimated for patient no. 10. Overall the average accuracy was 5.7 mm. The repeatability was determined as the difference in modulus between the two measurements in the supine position for the same patient recorded while carrying out the protocol. As a result, the range is between 0.2 mm for patient no. 20 and 1.5 cm for patient no. 7, whilst the average repeatability is 6.6 mm.

The perimeter, the area and the size function of the curve describing the torso cross-section have been calculated in every recorded position for each patient. Table 2 reports the average of these values in the supine position, which was recorded twice during the protocol. In a preliminary analysis the effect of the gestational age (hence the weight) was neglected and the lying position in which the minimum and maximum for each of the above three features were recorded for each subject was determined. As a result, the minimum circumference is estimated when the baby was lying on the right side for the majority of the subjects (32%), whilst the maximum was recorded for the baby lying supine (35%). A similar trend is captured for the area, being minimum mostly on the right side (29%), whilst the maximum is expected when the baby is 45 degrees to the right (26%) followed by the supine position (23%). An analogous outcome is obtained for the size function, which is minimum for the same number of patients lying 45 or 90 degrees to the right (29%) and maximum in the supine position (77%).

**Table 2:** Average of the measurements calculated in supine position.

Patient	Av. circumference (cm)	Av. area (cm$^2$)	Av. size function (cm)
1	34.2	91.3	1.1732
2	34.5	91.8	1.5200
3	33.6	87.4	1.6172
4	34.2	91.4	1.1758
5	34.1	88.6	1.9237
6	31.3	76.1	1.2843
7	36.2	99.0	2.2493
8	34.7	91.2	1.7609
9	32.8	82.4	1.7944
10	34.1	89.0	1.7190
11	31.6	77.8	1.2546
12	32.2	80.8	1.2100
13	32.7	82.4	1.5809
14	33.4	86.5	1.4299
15	35.3	96.4	1.5349
16	29.7	68.5	1.4322
17	30.1	70.4	1.2862
18	35.2	95.1	1.5543
19	25.6	51.5	0.7692
20	33.3	84.7	1.8129
21	33.3	85.8	1.2435
22	38.3	112.7	1.7557
23	32.1	80.8	1.1309
24	34.0	90.2	1.1696
25	32.7	83.1	1.2995
26	35.4	97.0	1.5228
27	31.1	75.6	1.0682
28	32.1	80.2	1.3978
29	32.5	81.7	1.3833
30	36.1	100.8	1.4408
31	28.0	61.4	1.0282
32	59.4	264.3	0.0387

In order to identify significant thoracic changes, statistical analyses have been carried out on the entire population and on a selection of four different gestational ages: 30 weeks, 33 weeks, 39 weeks and 41 weeks. The first two characterise the biomechanics of early pre-term babies whilst the latter two define the term subjects.

**Figure 2:**
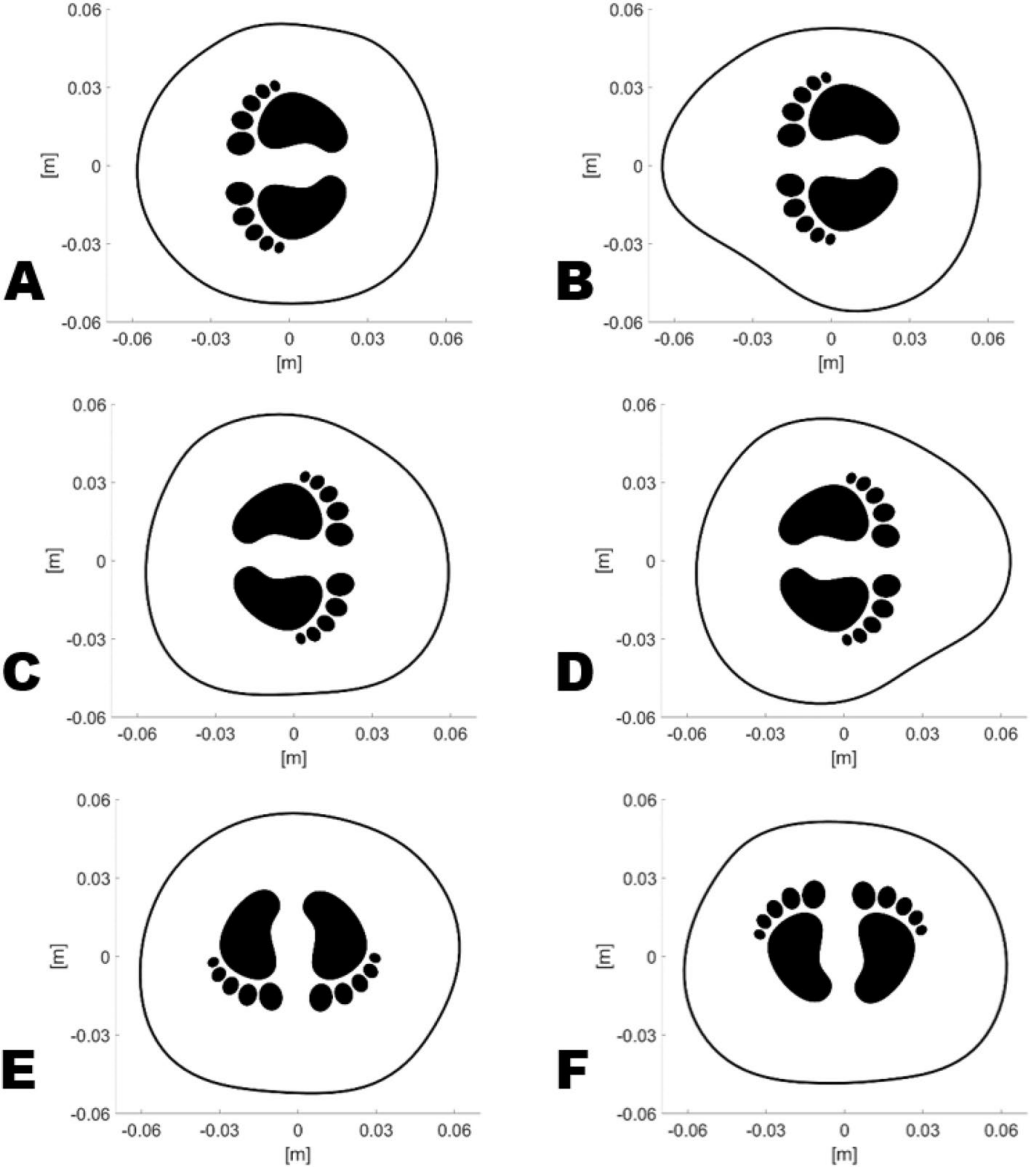
Comparison of the computed chest boundaries for subject no. 30. The baby was lying on her right side with her arms up (**A**) and down (**B**), on her left with her arms up (**C**) and down (**D**), prone (**E**) and supine (**F**).

The Anderson–Darling test established that the chest circumference and area values do not come from a normal distribution. Hence, the Kruskal–Wallis test has been adopted to determine if the samples come from the same population. Firstly, dividing the population into just two groups, preterm (less than 37 weeks of gestation) and term, highlights a considerable discrepancy in both the circumference and area. The selection based on the weeks of gestation is justified as the early pre-term subjects (30 weeks, 33 weeks) show anatomical dimensions significantly different from subjects born after 39 weeks of gestation (*p* values being 2.69e−06 for perimeter and 3.3e−06 for area). As the main interest of this study consists in identifying peculiar changes in the newborns’ chest caused by their lying position, four have been selected from the protocol: prone, 90 degrees to right, 90 degrees to left and supine. This selection is a result of the trends shown previously among all included subjects and aims to identify the main biomechanical changes. As an example the chest boundaries computed for patient no. 30 in different positions are shown in Fig. 2. Considering the position as unique grouping variable fails to detect any significant change in the circumference and area.

On the other hand, the computed size function values are normally distributed. Thus, the ANOVA test were performed. In this case the effect of the supine position alone generates shapes significantly different from lying both on the right and on the left side (*p* is 7.6e−05). Similarly, the prone position leads to statistically distinctive shapes compared to the lateral ones (*p* is 1.7e−04). Comparing only the prone to the supine highlights another substantial difference (*p* is 0.017), which is not evident once the four configurations are confronted.

A further multiway ANOVA investigated in detail the effect of the position and the week of gestation on the size function. As a result, for each of the selected week of gestation the values on the right are never statistically different from the ones on the left. Lying on the right side though produces shapes significantly different than the supine position for the subjects born at 39 weeks of gestation.

In order to rule out the effect of other factors on the observed change of chest shape, statistical analyses were carried out to test also the sex of the subjects as an independent variable. As a result, the sex is leading to no significant changes in terms of chest perimeter, area or size function even for the subjects born at 39 weeks of gestation. Lastly, testing for the effect of the ward where the subjects were cared for has not been thought sensible as all pre-term babies and only one newborn born at 39 weeks were in the NICU. Hence, no statistical consideration would be significant.

## Discussion

An unprecedented data collection about the boundary changes in the neonatal torso due to the lying position has been presented in this study. Although it is known to neonatologists that the chest of newborns is very compliant, no characterization was available. This phenomenon is highly relevant to the application of a patient interface EIT belt to monitor the lungs’ ventilation in newborns. The patient interface EIT belt in the CRADL project was suffering from loose fitting, with associated loss of electrode contact, in some positions for a number of subjects, hence the development of an electronic shape measuring belt for this investigation, which included 31 newborns and one child.

The accuracy (mean 5.7 mm) and repeatability (mean 6.6 mm) of the measuring belt have been assessed and judged satisfactory. It is worth noticing that the thickness of the encapsulation has been neglected. The thickness of the silicone rubber between the skin of the baby and the printed circuit board, where the accelerometers are placed, is 2 mm, which is controlled by the mould depth. Platinum cure silicone was used, which does not exhibit cure shrinkage. However, given the bespoke hand-made procedure it is difficult to establish any tiny dimensional change in this layer and to estimate the actual average thickness change. In addition, no compression test was carried out to quantify the deformation of such silicone encapsulation under the average weight of the subjects.

Figure 2 shows the chest boundaries, reconstructed in different positions, as an example of what happens in a typical term newborn. Firstly, the chest appears slightly compressible as the cross-sectional area is maximum in the prone position, decreases of 4.5% in the supine position and of 7% when lying 90 degrees to each side for this subject. Hence, it appears that the compression of the ribs, quite flexible at this age, plays a big role in the biomechanics of the torso. The distortion of the chest wall has been attributed to its increased compliance compared to the lung, to the incomplete ossification of the ribs and to the respiratory muscles unable to stabilize the chest wall^10^. Infants feature horizontally placed ribs compared to the downward-facing ones in adults, which allow a significant increase in both the anteroposterior and lateral diameters of the thorax when the diaphragm descends^5^. Nearly 2 years are needed for the infant rib-cage to mature into the adult configuration, stabilized by the external intercostal muscles, hence the breathing patterns compensate for the anatomic immaturity^5^.

**Figure 3:**
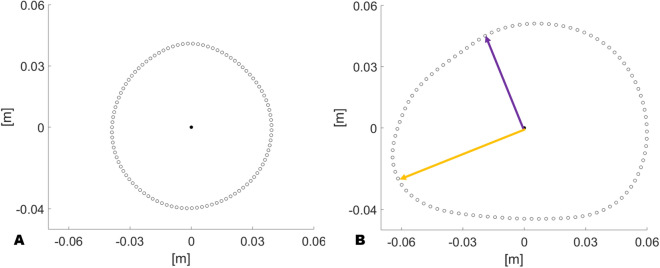
Comparison of the computed chest boundaries which feature the minimum (**A**) and the maximum (**B**) value of shape function. The purple arrow indicates the minimum distance from the centroid, whilst the orange one is the maximum distance.

A visible change in the morphology of the chest cross section is presented, especially when the lower arm is compressing the chest (Fig. 2B,D) compared to the other positions. The size function has been introduced to quantify and differentiate the shapes recorded in a way that the higher the value, the more irregular is the shape. In other words, the lower its value the more circular the shape looks like as shown in Fig. 3. The chest boundary of patient no. 19 (Fig. 3A) lying on his left side, being the lightest in weight among the recruited patient, looks almost like a regular circle. Differently, the boundary of patient no. 7 (Fig. 3B) lying supine appears asymmetric as the distance from the centroid ranges from 4.45 cm (purple arrow) to 6.66 cm (orange arrow).

**Figure 4:**
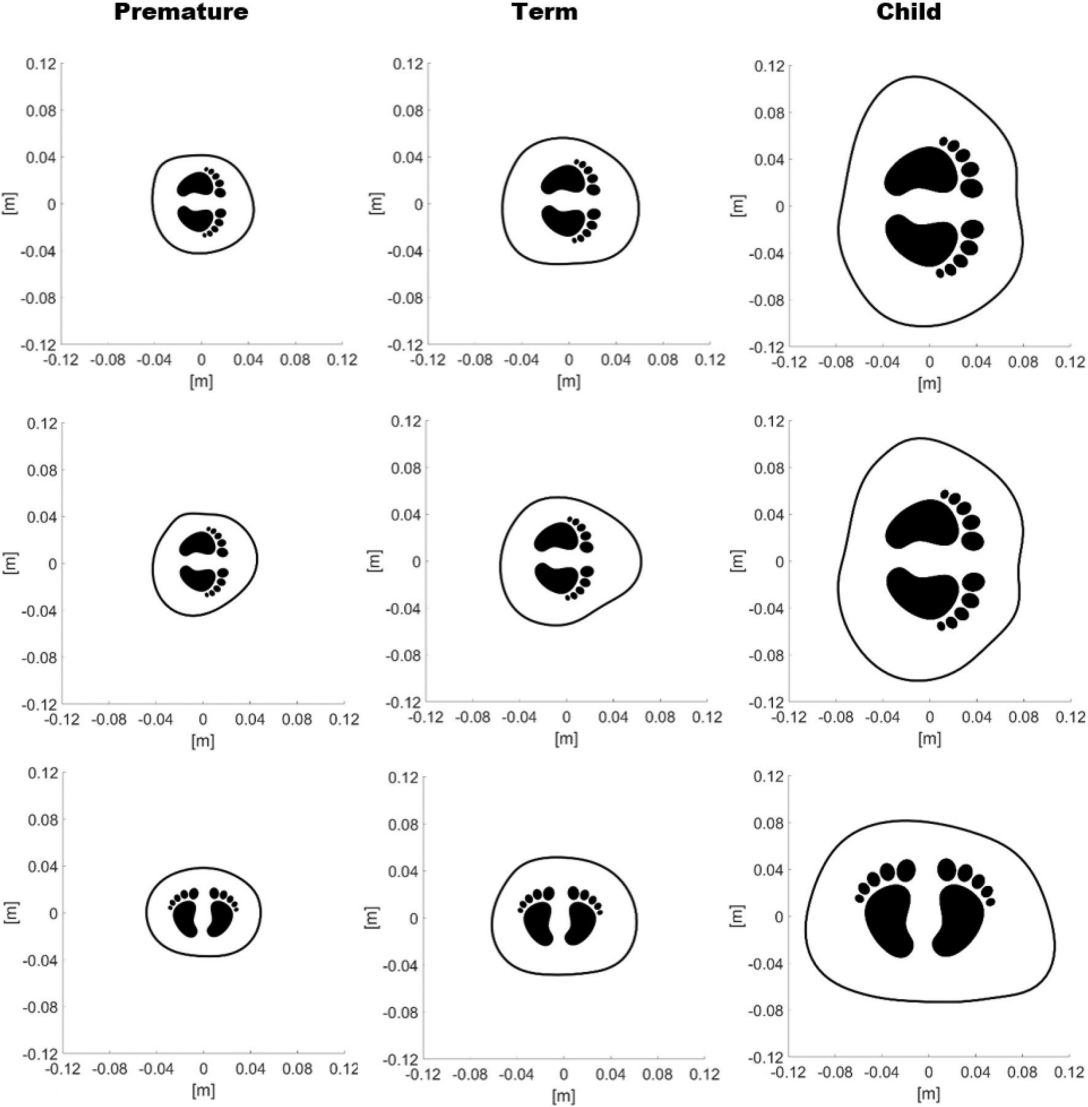
Evolution of the torso boundary computed for patient no. 31 (premature), patient no. 30 (term) and a child. On the top row the subjects are lying on their left side without compressing the torso with their arm. On the middle row their left arm lies between the mattress and the chest. On the bottom row the study subjects are lying supine.

One of the arms normally compresses the torso when lying on a side as newborns do not raise them. Hence the statistical analyses have taken into account only this physiological configuration.

An overall trend has been observed as the majority, approximately 30% of the subjects, exhibited minimum values of chest perimeters and area when lying on the right side. This effect could be explained by the fact that the highly compliant chest wall easily compresses more the right side, which unlike the left one does not feature the heart. Thus, the hypothesis emerges that the heart itself slightly constrains the deformation of the left side. The size function highlighted that a maximum value is observed in the supine position for 77% of the subjects, meaning that a greater scatter between the anteroposterior and lateral diameters leads to a more oval cross section.

The Anova test confirmed that considering only the effect of the four main configurations (prone, 90 degrees to right, 90 degrees to left and supine) the size functions computed are statistically distinctive comparing each of the lateral configurations to the prone or the supine ones. In addition, dividing the population into preterm and term and comparing the four configurations, the supine position is significantly different from the lateral ones only in the term subjects. A further Anova enquired if such difference is significant also in each group of gestational age. As a result, a remarkable difference between the size functions obtained on the right side compared to the supine position is estimated for the subjects born at 39 weeks of gestation. However, such a result could be biased by the highest number of samples (8 subjects) for the babies born after 39 weeks compared to the other subgroups. It is worth remembering that the present preliminary investigation is not a clinical study, thus the limited sample size. However, it has been noticed that the torso boundary deforms diversely in newborns based on their gestational age, thus perhaps because of the weight. The preterm subjects (30 and 33 weeks) exhibit a less pronounced deformation of the chest once placed on their side. Such an effect appears to be in contrast with the high compliance of the neonatal chest wall. However, these subjects feature an average weight of 2.042 kg against the mean of 3.524 kg at 39 weeks. Given the lower mass, a lower force is applied to the organs of the preterm babies, which module leads to limited deformations.

This work was able to test the measuring belt also on a child of 20 kg aged 6 years. The boundary obtained is compared to a premature newborn and a term one in Fig. 4. This illustration is novel, to the best of the authors’ knowledge, as it shows the maturation of the chest in young infants. On the top row it is clear that the sternum and the spine are visible only in the child. In the middle row, it is shown that the arm placed between the mattress and the chest generates a more pronounced deformation of the cross section in the term newborn. Such effect could be explained by the different weight, being 1.795 kg for the premature and 4.355 kg for the term, if compared with the other neonatal chest and by the different ossification if compared to the child. Finally, on the bottom row, although the dimensions are diverse for each subject, the boundary shapes appear comparable as gravity leads to an increased lateral diameter and to the disappearance of the concavity on the sternum even for the child.

This preliminary investigation intends to report an insight into the evolution of the chest in the infants and to demonstrate how the thorax changes in neonates. This information could be used by clinicians in medical practice, by engineers and designers in order to take into account such changes to improve the EIT belt fitting and to differentiate the reconstruction algorithm, which at the moment, to the best of the authors’ knowledge, neglects any deformation of the chest or compression of the lungs.

## Methods

### Study population and protocol

This study was carried out within the framework of the CRADL project, which has received funding from the European Union’s Horizon 2020 research and innovation programme. Following the approval of the Ethical Committee of the Northern Ostrobothnia Health Care District (ETTMK: 60/2018) 34 babies were asked to participate, 3 of whom the parents declined (1 in maternity ward, 2 in NICU). The study was performed in accordance with the relevant guidelines and regulations. After obtaining written informed consent of both parents, 31 newborns and 1 child were included in total from the Oulu University Hospital, Oulu, Finland. Informed consent from both parents was obtained for publication of the photographs in an online open-access publication.

At the beginning of the protocol, neonates were positioned supine on the mattress of an open incubator, above which the radiant warmer was already switched on for their comfort. Parents were welcomed to observe the whole procedure, which was carried out by the paediatricians among the authors of this study. Although statistically not relevant, the child was included in the study aiming to gain a preliminary insight about the thoracic change compared to the newborns and to assess the performance of the device on an older subject. The following details were acquired for each patient:

- Gestational age in weeks and days.
- Postnatal age in hours, weeks and days.
- Sex.
- Ward: maternity or NICU.
- Height at birth in cm.
- Weight at birth in cm.
- Head circumference at birth in cm.
- Chest circumference at the time of the study in cm.

The *Chest circumference* was evaluated below the nipple line by means of the standard measurement tape, similarly to what is done at birth for the Head. Successively, the electronic measuring belt was used to perform 7 measurements of the torso in 6 different positions as follows:

1. supine;
2. 45 degrees to right;
3. 90 degrees to right;
4. prone;
5. 45 degrees to left;
6. 90 degrees to left;
7. supine.

The repeated supine measurement was meant to assess the reliability of the records in comparison with the tape measure and to evaluate the sensitivity of the device.

### The electronic measuring belt

The device is designed to work as a smart tape. It features 32 high resolution 3-axis accelerometers ADXL313 (Analog Devices, Norwood, MA, USA) equally spaced every 16 mm. The electronics is perfectly insulated by means of a custom encapsulation made of a bio-compatible, platinum cure, medical grade silicone rubber, which prevents any possible current transfer to the skin of the baby. The encapsulation is soft, non-adhesive and has comfortable rounded edges. The device can be easily sanitized for each patient by wipes commonly used in the maternity wards. A prior soak test and a thorough explanation of the structure of the device was judged satisfactory by the ethical committee that approved the belt without additional safety testing. The belt was not classified as a medical device. The accelerometers in the measurement belt are powered (3.3 V) only by a laptop, sensors are then addressed and the data stored by an Arduino micro-controller connected to a laptop. The 32 accelerometers were assigned one of two I2C addresses and individually addressed via a 16 channel multiplexer (SparkFunElectronics, Boulder, CO, USA). The Arduino and multiplexer were housed in a transparent polycarbonate box (Hammond Manufacturing Ltd, Ontario, Canada). A live acquisition data link was established between the Arduino and MatLab via the laptop serial connection. Data were acquired by Arduino at 0.5 Hz and transmitted at 2 Mbps to the laptop. A control and visualisation interface was produced in MatLab to trigger the data collection, visualise the results and feedback on its completion. The belt is gently positioned around the chest and held in place by the clinician exactly like a standard measuring tape with no fastening adopted.

### The algorithm

**Figure 5:**
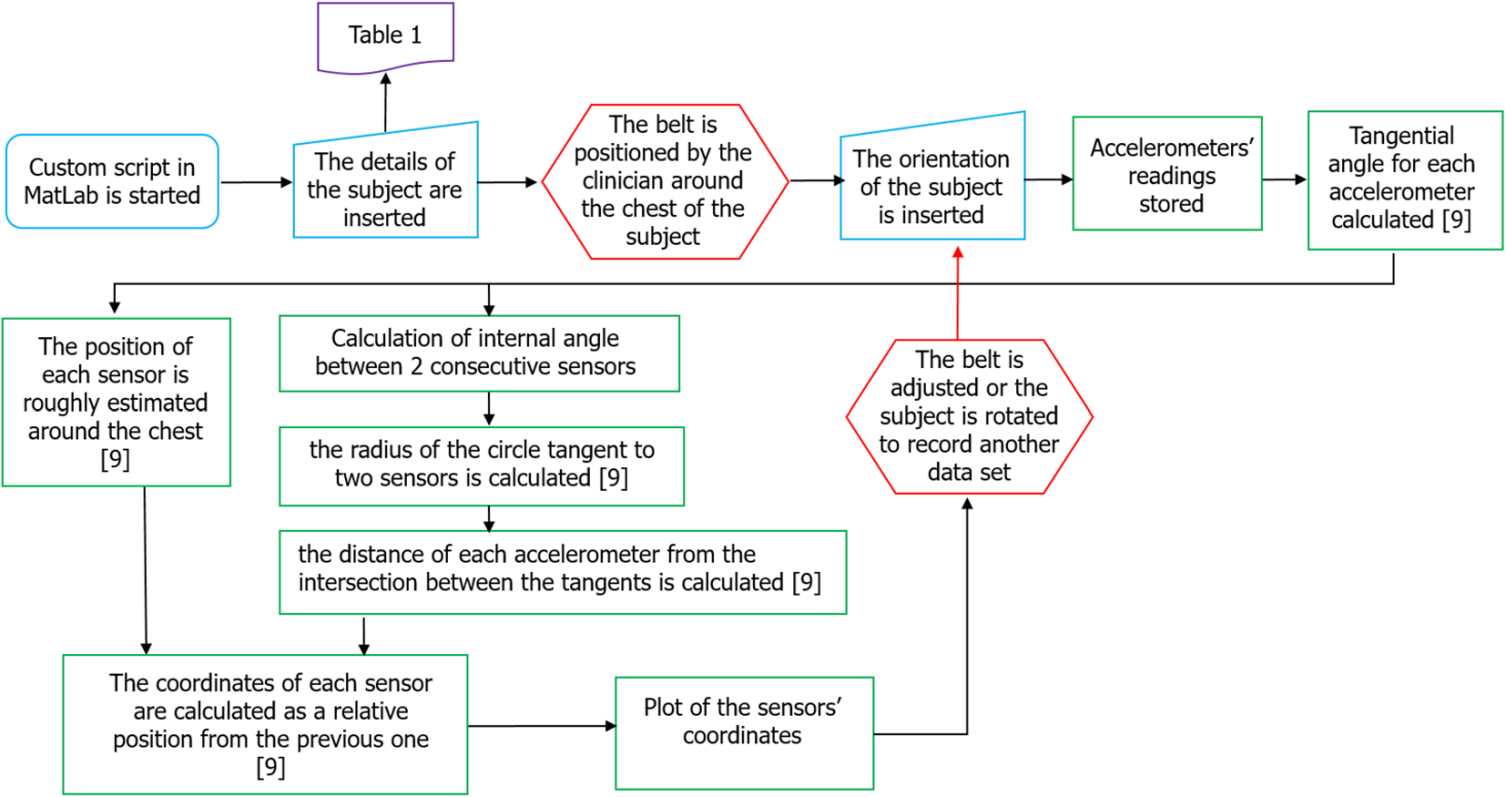
Algorithm steps (green) allowed to double check the data acquisition during the protocol, which was carried out by a clinician (red) supported by an engineer (blue). A previous work^9^ explains the core of the algorithm.

Once the belt is positioned around the chest of each subject, the measuring process is activated from the custom made script in MatLab (Mathworks, Nantick, MA, USA) on the laptop via a serial communication. A flowchart is reported in Fig. 5 to show how the algorithm and the protocol have been integrated.

The basics of the algorithm needed to acquire the torso shape via the accelerometers were reported in a previous work^9^. A series of prompts were added to insert in real time the details listed in the above detailed protocol of each patient before the actual recordings. The record of each position is instantaneously recorded, the cross-sectional area plotted as feedback and the data saved on the laptop.

As further novelty to the published study^9^, a custom-made function was implemented to take into account that the belt was designed to be long enough to accommodate the anatomical variability of all subjects. Therefore, the algorithm also automatically checks if there is a gap or overlap between sensor 1 and sensor 32 by estimating the distance between them. The distance between all the other accelerometers is iteratively estimated and sensors overlapping for a distance below 15.2 mm were removed from the plot of the cross-sectional chest area.

In case the baby suddenly moved or the belt was not positioned in a satisfactory configuration, further recordings were acquired. It is important to note that the sensors on the belt were calibrated prior to the study by placing the belt on a rigid rig oriented in 6 different configurations, accounting for the positive and negative components of the gravity vector recorded by the accelerometers along the axes X, Y and Z.

### Data analysis

Once the relative location of the accelerometers effectively delineating the torso boundary has been estimated, 100 equally spaced points in arc length were added by means of the *interparc* function in MatLab to obtain a better fitting. The arc length interpolation was successively carried out by the *interpclosed* function, which returns the perimeter, the area and the centroid of the curve describing the torso cross-section. Hence the coefficients of the piecewise polynomial interpolation are calculated and the area is estimated as an integral.

In order to quantify the change in shape of the reconstructed cross-sectional area, a size function has been introduced. The minimum and maximum distance from the centroid has been calculated for every recorded position. The size function is, therefore, defined as the difference between the maximum and the minimum of such distances (Fig. 3).

The statistical analyses were carried out firstly by checking the assumption that all sample populations are normally distributed. Such an assumption has been checked by the Anderson–Darling test, implemented in MatLab as *adtest*, which returns a test decision for the null hypothesis. In case the Anderson-Darling test fails to reject the null hypothesis at the default 5% significance level, the one-way Analysis of Variance (ANOVA), *anova1*, enables to find out whether different groups of an independent variable have different effects on the response variable. In the present study, the independent variable is, as an example, the lying position of the baby, while the perimeter and area are the response variables. In order to test the effect of multiple factors, such as the position and the gestational age, on the chest perimeter, area or size function a multiway ANOVA, *anovan*, has been employed. This test has been selected because of the unbalanced design, as the recruited subjects are not equally distributed among the different gestational ages. In case the Anderson–Darling test rejects the null hypothesis, the Kruskal–Wallis test, implemented in MatLab as *kruskalwallis*, compares the medians of the groups of data to determine if the samples come from the same population.
